# The Successful Management of Gangrenous Pneumococcal Infection in an Infant With Polysplenia Syndrome

**DOI:** 10.7759/cureus.41390

**Published:** 2023-07-05

**Authors:** Wallaa Garout

**Affiliations:** 1 Department of Pediatrics, King Abdulaziz University Hospital, Jeddah, SAU

**Keywords:** pneumonia, vaccination, streptococcus, sepsis, polysplenia

## Abstract

This study describes the successful management of gangrenous pneumococcal infection in an infant with polysplenia, a rare congenital malformation characterized by multiple aberrant nodules of splenic tissue. The patient, a three-month-old girl with congenital heart defects and incomplete vaccination, presented with fever, irritability, and oxygen desaturation, followed by erythematous skin changes. The diagnosis revealed sepsis caused by *Streptococcus pneumoniae*, accompanied by extensive gangrenous skin lesions and signs of disseminated intravascular coagulation. Polysplenia was incidentally discovered during radiological investigation. Aggressive resuscitation measures and prolonged antibiotic administration led to significant improvement, including regression of skin lesions. This case emphasizes the importance of timely immunization and parental awareness for infants with spleen-related congenital malformations. Screening for undiagnosed malformative defects in congenital heart disease patients can aid in early detection and prevention of life-threatening complications. The successful management demonstrates the critical role of pediatric intensive care units in treating severe systemic infections.

## Introduction

Polysplenia is a rare congenital malformation characterized by the presence of multiple (from two to 15) aberrant nodules of the splenic tissue [1]. Since it is due to embryonic anomalies in the lateralization, it is frequently associated with other congenital malformations, such as congenital heart defects (CHD), bilateral bilobation, or trilobation of the lungs, midline liver, right-sided stomach, and a degree of intestinal malrotation, forming a malformative syndrome usually referred to polysplenia syndrome [2].

Polysplenia results in impaired spleen function, which favors invasive infections with encapsulated bacteria, such as *Neisseria meningitidis*, *Haemophilus influenzae*,* *and *Streptococcus pneumoniae*. Therefore, infants with such conditions should receive the same preventive measures as asplenic or hyposplenic infants. These preventive measures include parents’ education, immunizations against *S. pneumoniae*, Hib, and *N. meningitidis*, long-term antibiotic prophylaxis, and aggressive management in case of infection suspicion [3]. However, as polysplenia can be an incidental finding, that may be diagnosed in adulthood when investigating other acute conditions, some infants may not benefit from adequate preventive strategies, and therefore, can develop severe infections by encapsulated bacteria [4].

Herein, we report a rare case of pneumococcal sepsis complicated with peripheral gangrenous lesions in an unvaccinated young girl against *S. pneumoniae*, who had CHD and undiagnosed polysplenia. Fortunately, after several days of aggressive management, the patient survived and had good evolution.

## Case presentation

A three-month-old girl was brought to our emergency department by her parents for one-day history of fever. She is known to have double inlet left ventricle (DILV), double outlet right ventricle (DORV), and pulmonary atresia (PA), for which she underwent patent ductus arteriosus (PDA) stenting at one month of age. Her home medications included furosemide 1 mg/kg/d OD, captopril 2 mg/kg/d TID, and aspirin 21 mg OD. The parents reported that she was in unusual health status before the presentation and then developed fever with irritability and excessive crying, which brought them to visit our hospital. The patient is a product of a full-term, booked pregnancy, complicated with gestational diabetes, premature rupture of membranes, and cesarean delivery. At birth, she started receiving prostaglandins infusions for bluish discoloration, and after performing cardiac ultrasound, the diagnosis of transposition of the great vessels with PA was made. The patient also had a history of septicemia at 15 days of age due to *Klebsiella pneumoniae* and *Staphylococcus aureus *successfully managed with antibacterial therapy. She received vaccination only at birth for unprecise reasons. Familial history was marked by first-degree consanguineous parents, with eight healthy elder siblings. No familial history of congenital malformations, genetic or metabolic disorders were reported.

On presentation, the patient was irritable, and cyanosed during crying, but not having respiratory distress. She was well hydrated with a capillary refill time of <2 s, and well responsive with a pediatric Glasgow Coma Scale (GSC) of 15/15. Her vital signs were as follows: temperature of 39.6°C, heart rate of 186 beats per minute (bpm), respiratory rate of 42/min, and oxygen saturation of 82% on room air. Her weight was 4.5 kg on the 5th percentile. Cardiovascular examination revealed an ejection systolic murmur on the left lower sternal border and a machinery murmur on the pulmonary area with no heave or thrill, which were likely attributed to the patient’s congenital condition. There was no rhinorrhea or inflamed tonsils. The lungs were clear on auscultation. The tympanic membrane had a normal aspect with no bulging or effusion and a well-visualized light reflex. Neurological examination was unremarkable with normal movements, posture and tone, and present reflexes. There were no rashes or skin anomalies.

Initial laboratory testing demonstrated the following findings: WBC levels of 17.67 K/µL, neutrophils of 13.85 K/µL, Hb of 12.1 g/dL, hematocrit (HCT) of 36.3%, mean corpuscular volume (MCV) of 80 fL, mean corpuscular hemoglobin (MCH) of 26.7 pg, platelets of 672 K/µL, blood pH of 7.39, partial pressure of carbon dioxide (PCO_2_) of 34.2 mmHg, bicarbonate (HCO_3_) of 21 mmHg, base excess of 3.7 mEq/L, and lactic acid level of 2.2 mmol/L.

Due to the patient’s young age, comorbidities, and abnormal laboratory findings, she was admitted to rule out sepsis by full screening for infection with adequate monitoring of the neurological and cardiorespiratory states. An empirical large-spectrum antibacterial therapy with vancomycin (20 mg/kg/dose every six hours) and ceftriaxone (100 mg/kg divided every 12 hours) was initiated after sending cultures from the peripheral blood, a catheter-obtained urine sample and a cerebrospinal fluid obtained by lumbar puncture, in addition to oxygen therapy with a target SpO_2_ ≥75%, strict input/output chart, and IV fluid 2/3 maintenance.

On the second day of admission, the patient had tachycardia reaching 180 bpm. She was still febrile at 39°C and developed mottling, which led to increasing her in vitro fertilization (IVF) to 1.5 maintenance. Lasix and captopril were administered with subsequent improvement in the heart rate and blood pressure. However, the patient started to have ecchymosis lesions and blood gas showed metabolic acidosis. Vancomycin was continued and ceftriaxone was replaced by imipenem (100 mg/kg/24 every six hours). Due to difficulty stabilizing the clinical status, the patient was referred to the pediatric intensive care unit (PICU) for strict monitoring.

After 11 hours of PICU admission, the patient’s status deteriorated, her temperature reached 40°C, and her skin erythema became more marked on the lower and upper limbs. A lumbar puncture was carried out and cerebrospinal fluid (CSF) culture came negative, ruling out meningococcal septicemia. However, blood culture revealed the presence of *Streptococcus pneumoniae *sensitivity, leading to the diagnosis of *S. pneumoniae* septicemia. At this time, a complete blood count (CBC) test showed a significant increase in the white blood cell count reaching 31 K/µL from 17.67 K/µL, neutrophils count to 19.60 K/µL from 13.85 K/µL, while platelets dropped to 34 K/µL from 672 K/µL and hemoglobin dropped to 9 g/dL from 12.1 g/dL requiring packed red blood cells transfusion of 15 mL/kg.

**Figure 1:**
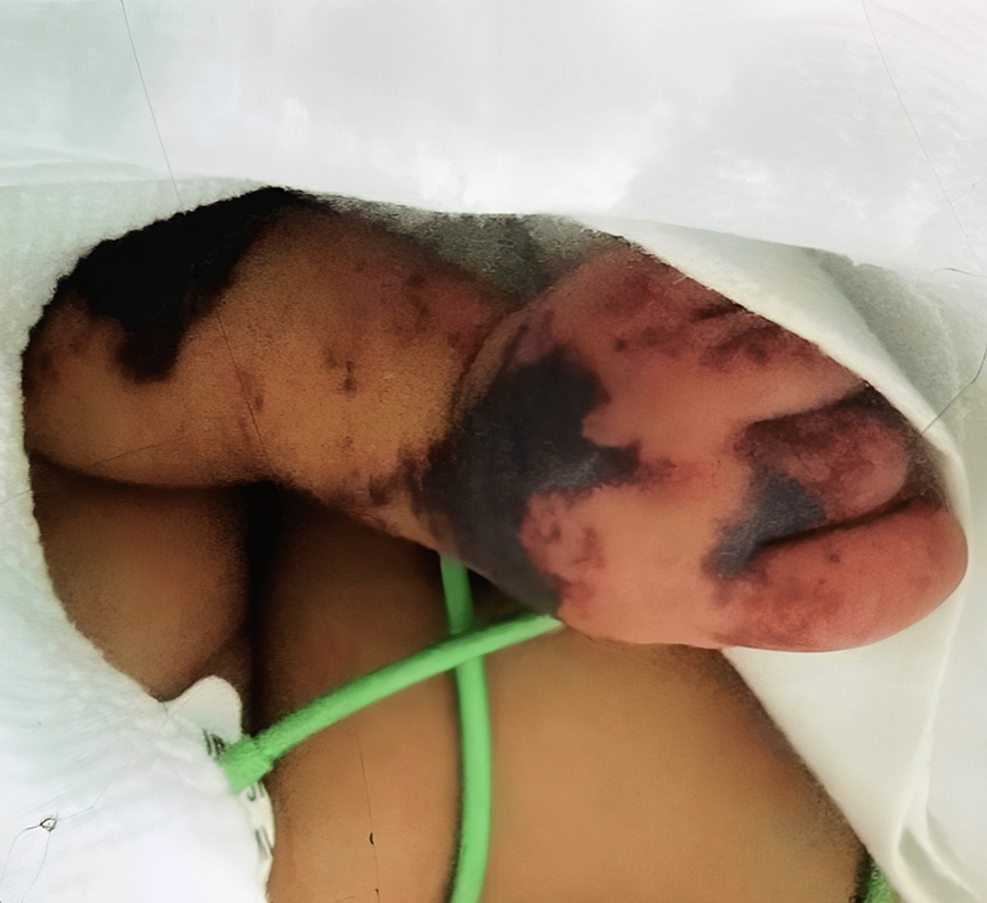
Gangrenous changes on the left arm on the sixth day of admission.

The patient’s condition became more critical with abrupt evolution of skin lesions to gangrenous patches that were more severe and painful in the digital areas, and rapidly extended to the pubis, perineal regions, then to most of the body (Figure 1). Additionally, the abdomen was distended with a palpable inferior hepatic border 3 cm below the costal margin.

**Figure 2:**
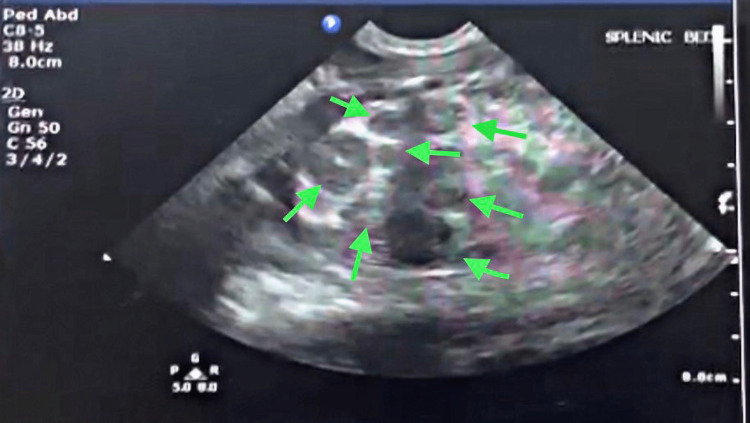
Abdominal ultrasound imaging of the left upper quadrant showing polysplenia.

Ultrasound of the abdomen was performed showing multiple well-defined round hypoechoic structures in the splenic bed, the largest measuring 1.3x1.5 cm, while the spleen itself was not visualized (Figure 2). These findings were suggestive of polysplenia. Doppler ultrasound of the upper and lower limbs did not reveal any arterial occlusions. Imipenem was continued and meropenem of 30 mg/kg/dose every eight hours was initiated instead of vancomycin.

On the sixth day of admission to the PICU, the patient exhibited tenderness in the lower limbs with a preponderance of symptoms in the anterolateral compartment of the right leg. Furthermore, ischemic patches that were circumferential in nature were observed in the left leg, accompanied by coldness in the extremities and weakened pulses. Referral to both vascular and plastic surgery was carried out, but interventional options were deemed unnecessary according to the surgical staff. Consultation with infectious disease specialists resulted in the recommendation to extend the patient's antibiotic therapy by an additional two weeks with ceftriaxone.

Consequently, signs indicative of disseminated intravascular coagulation were observed, including thrombocytopenia, decreased fibrinogen, and increased prothrombin time (PT) and partial thromboplastin time (PTT) (Table 1). The patient received fresh frozen plasma three times, platelet transfusion once, and cryoprecipitate once, resulting in the normalization of her coagulation profile and fibrinogen levels. Furthermore, the patient's general condition showed improvement, and her gangrenous lesions gradually regressed. After a 13-day hospitalization period, the patient was discharged with follow-up appointments in dermatology, cardiology, hematology, and pediatrics. She was also scheduled to receive her missed and programmed vaccines.

**Table 1:** Important blood studies progression. PLT: platelet count

	WBC (10^3^/µ)	Hb g/dL	PLT (10^3^/µ)	Fibrinogen 150-350 mg/dL	PT 11-14 S	PTT 29-40 S	INR 0.85-1.3 ratio
1st	17.67	12.1	672	109.5	32	69.9	2.9
2nd	23.74	9.5	125	149.7	31.8	49.8	2.3
3rd	29.85	10.8	70	164.4	26.3	42.4	1.6
4th	30.41	9	48	232.7	14.8	43.1	1.2
5th	30.33	13.7	34	293.6	14	46	1.1
6th	17.17	12.9	43	390.4	15.3	43	1.3
7th	16.63	11.5	89	384.1	15.5	43.1	0.9
8th	20.90	10.9	172	432.1	16.4	39	-
9th	21.11	12.4	254	-	-	33.2	-

## Discussion

In this study, we presented the case of a three-month-old female infant with a medical history of congenital heart disease, incomplete vaccination, and undiagnosed polysplenia, who exhibited fever, irritability, and oxygen desaturation on the first day of hospitalization, followed by erythematous skin changes on the second day. Subsequent diagnosis revealed sepsis caused by *S. pneumoniae*, which was accompanied by gangrenous transformation of skin lesions affecting much of the body, particularly the upper and lower limbs, as well as indicative signs of disseminated intravascular coagulation (DIC). During radiological investigation, the diagnosis of polysplenia was incidentally discovered. After aggressive resuscitation measures and prolonged antibiotic administration, the patient's condition improved considerably, with significant regression of skin lesions. While the occurrence of pneumococcal infection in polysplenic patients is rare in the literature, it is likely due to the rarity of polysplenia. Nonetheless, screening for this condition should be considered in infants with polymalformative defects, particularly those with CHD, as early diagnosis is crucial for preventing invasive infections.

In Saudi Arabia, it is standard protocol for all infants to receive their initial dose of anti-pneumococcal vaccine at two months of age, followed by subsequent doses at four months, six months, and 12 months of age, comprising the second, third, and fourth doses, respectively [5]. The vaccine utilized in Saudi Arabia is PCV13, which consists of a purified capsular polysaccharide of 13 serotypes, including 1, 3, 4, 5, 6A, 6B, 7F, 9V, 14, 19A, 19F, 18C, and 23F, conjugated to the diphtheria nontoxic protein, CRM197. This vaccine offers protection against 46% of vaccine-type pneumococcal pneumonia cases, 45% of vaccine-type non-bacteremic pneumococcal pneumonia cases, and 75% of vaccine-type invasive pneumococcal disease cases [6].

This particular case serves to illustrate that vaccination delays are still occurring in Saudi Arabia, potentially attributable to parental hesitancy or restricted access to immunization centers. A cross-sectional study conducted in Saudi Arabia that surveyed 500 parents found that 20% of the participants exhibited hesitancy toward vaccinating their children. Consequently, 36% of the children belonging to hesitant parents had not received full vaccination. The primary reason for vaccine hesitancy was reported to be concerns regarding the safety of vaccines [7]. A separate study examining preschool children residing in Jeddah found that 24.2% of them had experienced vaccination delays, primarily resulting from difficulties in traveling to vaccination appointments at the time of scheduled vaccinations (21.3%), limited availability of the vaccine in healthcare facilities (15.5%), and transportation issues (14.1%) [8]. The sudden progression of the patient's condition highlights the potentially serious consequences that may arise from delays in administering the *S. pneumoniae* vaccination, particularly in infants with underlying comorbidities, such as CHD. The presence of CHD in our patient represented a significant risk factor for severe infection, as previous studies have established an increased incidence of culture-proven sepsis in infants with CHD [9].

Cutaneous gangrene is a rare complication of pneumococcal sepsis that was reported in 6% of cases [10]. In modern times, only a few cases of this condition were published with majority of the affected patients being asplenic, unvaccinated, or immunocompromised [11-14]. Similar to meningococcal gangrene, gangrene due to *S. pneumoniae *tends to be symmetrical and affects predominantly the extremities with signs of DIC including either thrombocytopenia below 50,000/mm^3^, abnormal coagulation studies, or both [10]. The underlying pathophysiology of purpura fulminans in *S. pneumoniae *sepsis is thought to be primarily related to peripheral vasoconstriction, rather than the dermal microvascular thrombosis observed in cases of purpura fulminans associated with meningococcal sepsis, which is primarily a result of prothrombotic events [10,15]. Another difference between purpura fulminans induced by *S. pneumoniae *and *N. meningitides *is the lesser frequency of hypotension and hypofibrinogenemia in the former, which may explain the better prognosis in our case [16].

Furthermore, this case underscores the significant impact of appropriate resuscitation strategies on the management and prognosis of sepsis-related complications in critically ill patients admitted to PICU, even in the presence of extensive cutaneous gangrene. These measures encompass early and continuous administration of broad-spectrum antibiotics, vigilant monitoring and maintenance of optimal oxygen saturation and hydration status, and prompt but judicious correction of coagulopathy if concurrent disseminated intravascular coagulation is present.

## Conclusions

The current study highlights the heightened susceptibility of infants with congenital malformations of the spleen, especially those with delayed vaccination, to invasive pneumococcal infections. This emphasizes the need to increase parental awareness about the criticality of timely immunization in this patient population, in order to prevent vaccine delay or hesitancy. Additionally, screening for undiagnosed malformative defects in infants with CHD may be a valuable approach to identifying severe conditions, such as polysplenia, and preventing their potentially life-threatening complications. Furthermore, our study demonstrates that early and aggressive resuscitation measures, with close monitoring and adaptive strategies, can effectively manage severe and extensive cutaneous gangrene resulting from bacterial sepsis. These findings underscore the critical role of PICU in altering the outcomes of severe systemic infections.
